# Characterization of intrinsic properties of cingulate pyramidal neurons in adult mice after nerve injury

**DOI:** 10.1186/1744-8069-5-73

**Published:** 2009-12-16

**Authors:** Xiao-Yan Cao, Hui Xu, Long-Jun Wu, Xiang-Yao Li, Tao Chen, Min Zhuo

**Affiliations:** 1 Department of Physiology, Faculty of Medicine, University of Toronto, Medical Science Building, 1 King's College Circle, Toronto, Ontario M5S 1A8, Canada; 2 Department of Brain and Cognitive Sciences, Seoul National University, Seoul 151-746, Korea

## Abstract

The anterior cingulate cortex (ACC) is important for cognitive and sensory functions including memory and chronic pain. Glutamatergic excitatory synaptic transmission undergo long-term potentiation in ACC pyramidal cells after peripheral injury. Less information is available for the possible long-term changes in neuronal action potentials or intrinsic properties. In the present study, we characterized cingulate pyramidal cells in the layer II/III of the ACC in adult mice. We then examined possible long-term changes in intrinsic properties of the ACC pyramidal cells after peripheral nerve injury. In the control mice, we found that there are three major types of pyramidal cells according to their action potential firing pattern: (i) regular spiking (RS) cells (24.7%), intrinsic bursting (IB) cells (30.9%), and intermediate (IM) cells (44.4%). In a state of neuropathic pain, the population distribution (RS: 21.3%; IB: 31.2%; IM: 47.5%) and the single action potential properties of these three groups were indistinguishable from those in control mice. However, for repetitive action potentials, IM cells from neuropathic pain animals showed higher initial firing frequency with no change for the properties of RS and IB neurons from neuropathic pain mice. The present results provide the first evidence that, in addition to synaptic potentiation reported previously, peripheral nerve injury produces long-term plastic changes in the action potentials of cingulate pyramidal neurons in a cell type-specific manner.

## Background

The anterior cingulate cortex (ACC) is important for the affective and emotional component of physiological and pathological pain [1-5]. Brain imaging and electrophysiological studies have shown that the ACC responds to painful stimuli in humans [4,6,7] and nociceptive stimuli in animals as well [8-10]. Activation of neurons in the ACC produced pain-like aversive behaviors or fear memory, while inhibition of excitatory transmission produced the blockade of pain-aversive learning or analgesic effects [1,5,11,12]. Moreover, there are long-term changes in synaptic plasticity and transmitter release in the ACC in chronic pain conditions: amputation causes long-term facilitation of local stimulation-induced ACC synaptic responses and specific loss of long-term synaptic depression [13,14]; peripheral inflammation of the hind paw with complete Freund's adjuvant in adult mice causes the upregulation of the postsynaptic NMDA receptor NR2B subunit [15]. Furthermore, increased presynaptic glutamate releases were found in ACC neurons after inflammation [16] or nerve injury [17].

Neuropathic pain occurs as a consequence of the injury to peripheral or central nervous system. Symptoms include spontaneous pain, abnormal hypersensitivity to innocuous touch (allodynia) and to noxious mechanical or thermal stimulation (hyperalgesia) [18,19]. Clinical neuropathic pain can arise from a variety of different disease states (e.g., diabetic neuropathy, trigeminal neuralgia, postherpetic neuralgia, AIDS) or traumatic injuries, nerve compression, or chemotherapy [20]. Long-term plastic changes along the sensory pathways are suggested to contribute to the neuropathic pain, including the peripheral nociceptors, spinal dorsal horn, subcortical areas and cortical areas [21-23]. Of particular interest here is that the ACC is undergoing dramatic changes under chronic pain conditions [21,22]. However, little is known about the intrinsic electrophysiological properties of ACC neurons and possible changes in intrinsic properties after nerve injury. One major hypothesis is that altered neuronal excitability after peripheral nerve injury may contribute to the plastic changes of ACC neurons under chronic pain conditions. To test the idea, by using the whole-cell patch-clamp recordings in slices under current clamp mode, we examined the firing activity of ACC neurons in a mouse model of neuropathic pain reported previously [24].

## Results

**Figure 1:**
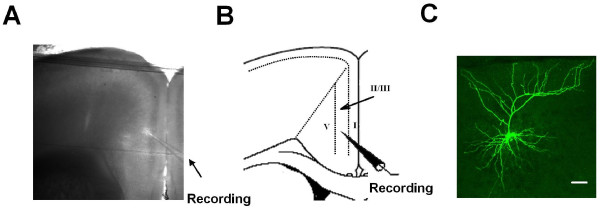
**Whole cell patch clamp recordings were made in the anterior cingulate cortex (ACC)**. A, Representative coronal section showing the placement of a whole-cell patch recording in a cingulate slice; B, Diagram representation of the location of the recorded neurons in layer II/III; C, Photomicrograph of a representative biocytin--labeled layer II/III ACC pyramidal neuron as visualized with confocal laser scanning microscopy.

We performed whole-cell patch-clamp recordings for the layer II/III neurons in the ACC of adult mice (Fig 1A, B). We decided to focus on Layer II-III cells, because that (i) neurons in this regions receive sensory inputs from thalamus [25,26]; (ii) our previous studies showed that synapses in Layer II-III undergo plastic changes after LTP induction [15,16,21,22,27] or peripheral nerve injury [17]. After a stable recording was obtained, these neurons were electrophysiologically characterized and simultaneously injected with biocytin for histochemical processing and morphological analysis (Fig. 1C). All neurons included in the present study are pyramidal cells, of which 61 pyramidal cells were from slices of neuropathic pain mice (n = 12 mice) and 81 pyramidal cells were from slices of non-operated (n = 10 mice) and sham-operated mice (n = 6 mice).

### General characteristics of pyramidal cells in layer II/III of ACC

**Figure 2:**
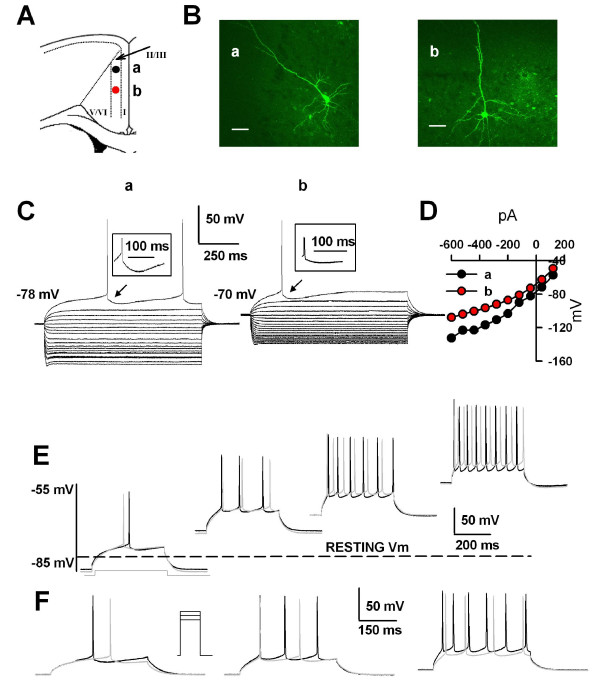
**Characteristics of the typical RS neurons in the ACC**. A, Schematic representation of the location of the recorded neurons (a and b); B, Biocytin profiles of two representative ACC RS pyramidal neurons as visualized with confocal laser scanning microscopy. Scale bar = 50 μm. C, Superimposed current-clamp traces in response to a series of intracellular current pulses (1000 ms, 80 pA per step). The neurons exhibited an AHP with a slow component. Note that an obvious deflection was observed during the hyperpolarizing phase; D, I-V plots constructed from the values of traces shown in (C) displayed linearity in the membrane voltage range between -65 and -90 mV; E, Traces evoked by the same current injections (400 ms, 100 pA) as the neurons was depolarized from -85 to -50 mV. The more the cells were depolarized, the more the action potentials were elicited by the same depolarizing pulse. Each spike was followed by a sAHP that lasted for tens of milliseconds. Note that the black traces were for cell (a) and gray traces for cell (b); F, Action potential trains were evoked by 400 ms current injection of 160, 180 and 200 pA from the holding potential of -70 mV. Note the increase in the frequency of action potential discharge with increasing current injections. Note that the black traces were fro cell (a) and gray traces for cell (b).

**Figure 3:**
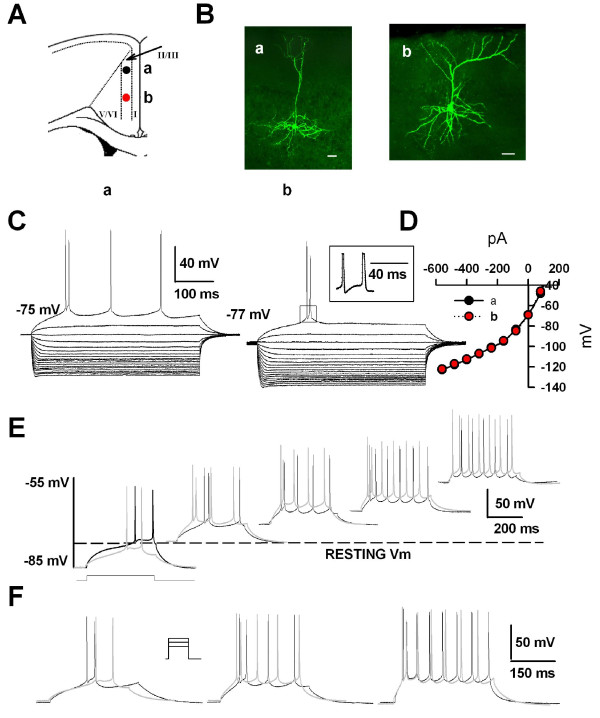
**Electrophysiological characteristics of typical IB ACC neurons**. A, Schematic representation of the location of the recorded neurons (a and b); B, Biocytin profiles of two representative IB ACC pyramidal neurons as visualized with confocal laser scanning microscopy. Scale bar = 50 μm; C, Superimposed current-clamp traces in response to a series of intracellular current pulses (1000 ms, 80 pA per step). The neurons exhibited an AHP with a big afterdepolarization (ADP), which could facilitate an action potential and therefore, formed a burst response; D, I-V plots constructed from the values of traces shown in (C) displayed a linear response in the voltage range between -60 and -85 mV; E, Traces evoked by the same current injections (400 ms, 100 pA) as the neurons were depolarized from -85 to -50 mV. When held at a hyperpolarized membrane potential, the burst response was evoked in response to the depolarizing current injections. By contrast, with gradual depolarization (V_m _≥ -65 mV) (left to right), a train of single spikes was evoked in response to the same current pulse and the burst was inactivated. The dotted line denotes the resting membrane potential. Note that the black traces were for cell (a) and gray traces for cell (b); F, Action potential trains were evoked by 400 ms current injection of 60, 80 and 100 pA from the holding potential of -70 mV. Note that a lower current intensity evoked an initial doublet of action potentials while higher current evoked an initial doublet of action potentials followed by a regular discharge. Note that the black traces were for cell (a) and gray traces for cell (b).

**Figure 4:**
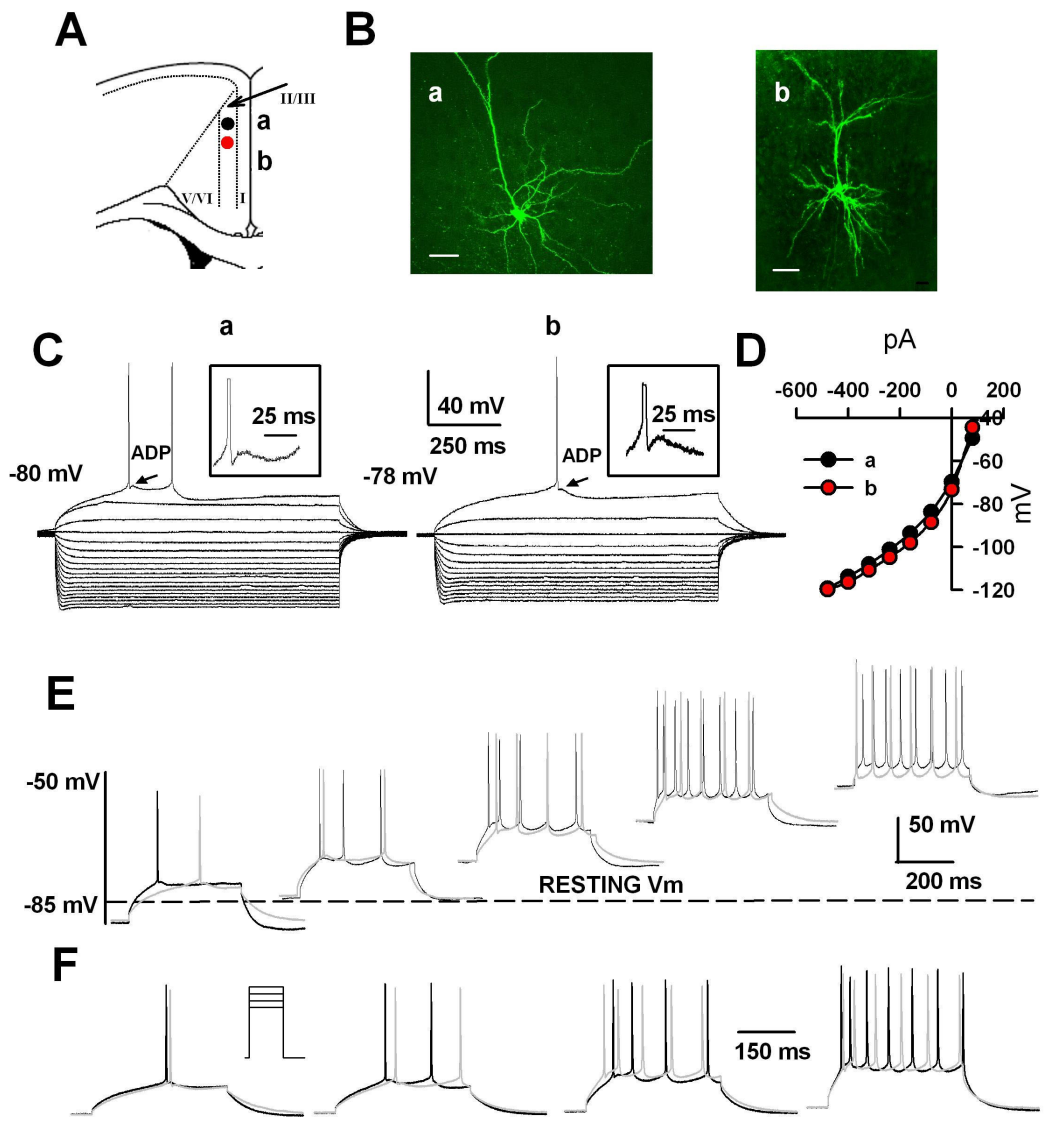
**Electrophysiological characteristics of the IM cells**. A, Schematic representation of the location of the recorded neurons (a and b); B, Biocytin profiles of those two representative IM ACC pyramidal neurons as visualized with confocal laser scanning microscopy. Scale bar = 50 μm. C, Superimposed current-clamp traces in response to a series of intracellular current pulses (1000 ms, 80 pA per step). The neurons exhibited an AHP with an early fast and a delayed slow component. Fast and slow components were intercalated by an afterdepolarization (ADP). D, I-V plots constructed from the values of traces shown in (C) showed a linear response in the voltage range between -65 and -85 mV. E, Traces evoked by the same current injections (400 ms, 100 pA) as the neurons were depolarized from -85 to -50 mV. Note that on no occasion did the IM neurons fire in bursts. The black traces were for cell (a) and gray traces for cell (b); F, Action potential trains were evoked by 400 ms current injection of 120, 140, 160 and 180 pA from the holding potential of -70 mV. Note that only the first spike was followed by an ADP. Traces reflect spike frequency adaptation in evoked action potential trains. The black traces were for cell (a) and gray traces for cell (b).

In many excitable cells, action potentials are followed by after-potentials that regulate the excitability of the cell for periods ranging from a few milliseconds to several seconds [28,29]. In the ACC neurons, we found different patterns of afterhyperpolarization (AHPs), with or without afterdepolarization (ADP), followed individual action potentials. Three distinct groups of pyramidal neurons in layers II/III of the ACC were classified based on their evoked firing patterns in response to depolarizing pulses and characteristics of the AHPs followed the action potentials (see Figs. 2, 3 and 4 for examples). They have been called regular spiking (RS), AHPs without ADP; intermediate (IM), AHPs with ADP; and intrinsic bursting (IB) cells, AHPs with both ADP and the burst activity triggered by the ADP. For morphological properties of these cells (Fig. 2B, 3B and 4B for examples), we found that all pyramidal cells had a prominent apical dendrite, which ascended toward the superficial layers, gave off some branches usually within layer I and formed apical tufts. Their basal dendrites were mainly located within the same layer as the soma. In most cases, the main axon was directed toward the deeper layers, such as V and VI.

### Regular Spiking (RS) neuron

Twenty ACC neurons (24.7%) were classified as RS cells. The averaged resting potential of RS cells was -74.4 ± 2.3 mV and their averaged action potential threshold was -33.4 ± 1.0 mV (see Table 1). The voltage-current relationships of these typical RS neurons were linear at membrane potentials between -65 and -90 mV (Fig. 2D), yielding a mean slope resistance of 120.1 ± 13.6 MΩ. In response to an injection of a suprathreshold depolarizing current pulse, RS neurons typically fired single spikes followed by a slow component and an obvious deflection of fAHP and ADP (<0.5 mV) during the hyperpolarizing phase (Fig. 2C). The mean amplitude for the sAHP was -12.5 ± 0.7 mV (from -6.8 mV to -16.2 mV) and the time to peak of the sAHP was 73.3 ± 7.4 ms (from 43.3 to 125.7 ms). In RS cells, a just suprathreshold depolarizing current could only evoke single action potentials and no spike bursting complex could be evoked even with carefully grading of injected current intensity near the threshold. Gradually holding the cell to more positive potentials, RS cells fired more spikes in response to the same depolarizing pulses with longer interspike interval between subsequent spikes (Fig. 2E). At the resting membrane potential (RMP), stronger depolarization also triggered a train of single spikes with marked spike frequency adaptation (Fig. 2F).

**Table 1:** Summary of basic electrophysiological and morphological parameters of ACC layer II/III pyramidal neurons in control and neuropathic pain mice.

	RS cells		IM cells		IB cells	
	control	Neuropathic pain	control	Neuropathic pain	control	Neuropathic pain
	(n = 20)	(n = 13)	(n = 36)	(n = 29)	(n = 25)	(n = 19)
RMP, mV	-74.4 ± 2.3	-72.4 ± 2.2	-70.8 ± 1.8	-70.7 ± 3.0	-74.7 ± 1.1	-70.5 ± 1.4
AP threshold, mV	-34.4 ± 1.0	-37.5 ± 1.0	-35.2 ± 0.6	-33.3 ± 0.8	-36.0 ± 0.7	-35.4 ± 1.1
Input Resistance, mΩ	120.1 ± 13.6	146.8 ± 13.6	122.1 ± 12.1	133.3 ± 16.6	129.5 ± 8.0^a^	130.2 ± 9.8
Spike Height, mV	94.0 ± 2.7	88.3 ± 3.6	92.9 ± 1.4	93.6 ± 1.9	93.3 ± 1.3	89.4 ± 4.0
Spike Half Width, ms	1.66 ± 0.07	1.87 ± 0.17	1.46 ± 0.05^a^	1.57 ± 0.02	1.43 ± 0.04^b^	1.40 ± 0.07
fAHP Amplitude, mV	--------	--------	-7.0 ± 0.6	-8.2 ± 0.7	-6.8 ± 0.4	-6.4 ± 0.8
Time of fAHP, (ms)	--------	--------	5.0 ± 0.2	5.3 ± 0.4	4.0 ± 0.3^d^	3.9 ± 0.5
sAHP Amplitude, mV	-12.5 ± 0.7	-11.5 ± 0.8	-9.6 ± 1.6	-11.6 ± 0.8	-12.1 ± 0.8	-9.8 ± 1.0
Time of sAHP, ms	73.3 ± 7.4	70.8 ± 8.2	70.0 ± 3.9	56.5 ± 11.8	89.4 ± 7.6	71.2 ± 5.4
ADP, mV	--------	--------	1.5 ± 0.2^c^	1.3 ± 0.2	3.43 ± 0.40^c, e^	2.4 ± 0.4
Rise Time, ms	0.60 ± 0.03	0.66 ± 0.04	0.57 ± 0.02	0.54 ± 0.01	0.58 ± 0.02	0.56 ± 0.02
Rise Slope, mV/ms	132.4 ± 7.3	113.6 ± 11.4	137.0 ± 6.2	141.0 ± 3.5	136.9 ± 4.8	139.4 ± 7.8
Decay Time, ms	2.09 ± 1.12	2.01 ± 0.18	1.63 ± 0.06^b^	1.96 ± 0.12	1.53 ± 0.08^c^	1.44 ± 0.09
Decay Slope, mV/ms	-36.6 ± 2.9	-37.3 ± 4.9	-45.7 ± 1.9^a^	-37.9 ± 3.1	-50.9 ± 3.2^a^	-52.9 ± 4.5
Rheobase, nA	112.3 ± 8.5	106.9 ± 29.0	97.4 ± 7.6	80.3 ± 21.1	75.8 ± 3.7^b^	77.5 ± 19.5

Values are means ± SEM; *n *is number of cells. ACC, anterior cingulate cortex; RMP, resting membrane potential; fAHP, fast afterhyperpolarization; sAHP, slow afterhyperpolarization; ADP, afterdepolarization.

^a^Significantly different from RS cells at *P *< 0.05.

^b^Significantly different from RS cells at *P *< 0.01.

^c^Significantly different from RS cells at *P *< 0.001.

^d^Significantly different from IM cells at *P *< 0.01.

^e^Significantly different from IM cells at *P *< 0.001.

### Intrinsic bursting (IB) neuron

Twenty five ACC neurons (30.9%) were classified as IB cells. The passive membrane properties of bursting cells were similar to those of the RS and IM cells (Table 1). IB neurons had a mean RMP of -74.7 ± 1.1 mV, a mean action potential threshold of -36.0 ± 0.7 mV and a linear voltage-current relationship between -60 and -85 mV with a mean slope resistance of 129.5 ± 8.0 MΩ (Table 1). When activated from the resting membrane potential by an intracellular current pulse, the action potential was followed by an AHP with a big ADP (n = 14, 3.4 ± 0.4 mV), which can facilitate an action potential and therefore, form a burst response (Fig. 3C, F). The ability of IB cells to generate bursting is the primary physiological properties that distinguish bursting cells from the two other types. The character of the burst was not always all or none. Some cells (n = 14), in response to a just superthreshold current injection, displayed single spikes with prominent ADP. However, a burst can always be evoked by slightly increasing the current intensity. When the membrane potential was gradually depolarized, pulses of current could only elicit regular spaced spike (Fig. 3E), suggesting that, in IB cells, the generation of burst during a stimulus was dependent upon the previous voltage of the membrane. At the constant membrane potential of -70 mV, lower current intensity elicited initial doublet of action potentials, while higher current evoked an initial burst followed by a train of current potential (Fig. 3F).

### Intermediate (IM) neuron

**Figure 5:**
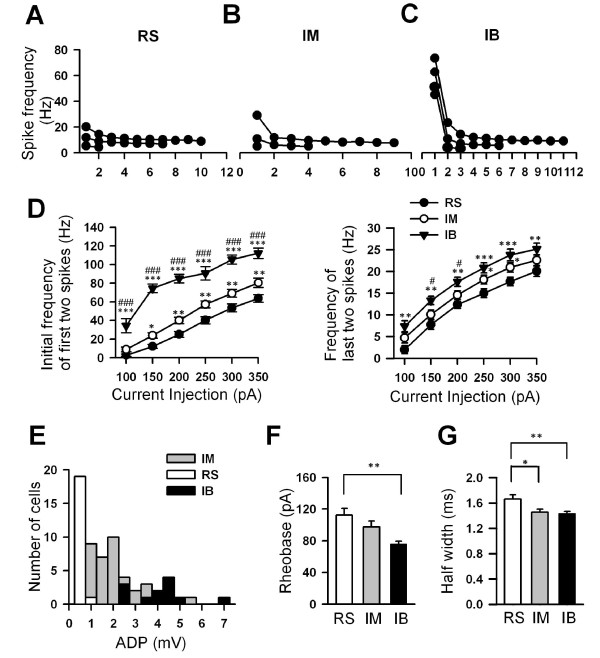
**Differential intrinsic membrane properties between RS, IM and IB pyramidal cells**. A-C, Plots of spike discharge frequencies for each interspike interval during the current injection illustrated in Figures 2F, 3F, 4F for RS, IM and IB cells. After the stimulating current reached spike threshold in each of the 3 representative cells, 3-4 current steps, each of 40 pA were applied. Note that at just above the rheobase, IB cell fired an initial burst with two APs at up to 50 Hz, whereas RS cell and IM cells fired APs regularly at ~5 Hz; D, Plots of the mean spike discharge frequencies, measured from the 1^st ^interspike interval (left) and the last interspike interval (right) for RS (n = 20), IM (n = 36) and IB (n = 25) cells, at different stimulation intensities of depolarizing current (100--350 pA, 400 ms). In response to the same intensities of current RS generated spikes at the lowest frequency and degree of initial frequency moderately increased with increase in current intensity. IB generated an initial doublet of action potentials with highest frequency, which increased with increase in current intensity. The initial frequency of the spikes for IM was in between. Values are given as the mean ± S.E.M. *P < 0.05, significantly different from RS cells; **P < 0.01, significantly different from RS cells; ***P < 0.001, significantly different from RS cells; #P < 0.05, significantly different from IM cells; ###P < 0.001, significantly different from IM cells; two-way ANOVA. E, Histogram showing the distribution of the amplitude of the ADP. Black bars indicate the ADP of IB neurons (n = 14). Gray and white bars indicate the ADP of IM (n = 36) and RS (n = 20) neurons respectively. F, Histogram reflects the rheobase for RS (n = 20), IM (n = 36) and IB (n = 25) cells (**P < 0.01 based on one way ANOVA). Values are given as the mean ± SEM. (G) Histogram summary data of the half widths of the RS (n = 20), IM (n = 36) and IB (n = 25) cells (*P < 0.05 and **P < 0.01 based on one way ANOVA).

By far the most frequently recorded type of neurons in layers II/III of the ACC (n = 36, 44.4%) was classified as IM. The reason why these cells are called intermediate is that their electrophysiological characteristics were intermediate between RS and IB cells (Fig. 4). For the intrinsic membrane properties of IM cells, there was no significant difference from that of typical RS cells (Table 1). The averaged RMP of IM cells was -70.8 ± 1.8 mV. The mean input resistance was 122.1 ± 12.1 MΩ and the mean action potential threshold was -35.2 ± 0.6 mV. In response to a just superthreshold current, the action potential of these neurons was followed by a fAHP which was subsequently followed by a small ADP (Fig. 5E; n = 36, 1.5 ± 0.2 mV) and a subsequent sAHP (Fig. 4C), a key facture that distinguish the IM cells from RS cells. The presence of ADP is considered to indicate a tendency for a cell to fire in bursts. However, IM cells did not generate spike bursts when RMP was manipulated over a wide range (from -85 mV to -55 mV) with tonic current injection. At more depolarized potentials (> -60 mV), the fAHP and the ADP diminished and the spike trains showed a progressive adaptation in frequency, which was indistinguishable from RS cells (Fig. 4E). At the RMP, in response to different intensities of current pulses, cells fired trains of spikes with the first spike followed by the postspike fAHP, ADP, and sAHP (Fig. 4F).

### Differential electrophysiological properties among RS, IM and IB pyramidal cells

Although RS, IM and IB pyramidal cells showed similarities in many prospects of electrophysiological properties, such as resting membrane potential, membrane resistance, current threshold and action potential amplitude, these neurons exhibited significant differences in some of the intrinsic properties. In general, those cells had the lowest membrane excitability for RS cells and the highest membrane excitability for IB cells with IM cells in between. To evoke action potential, RS cells needed significantly higher mean intensity of current injection (112.3 ± 8.5 pA) as compared with IB cells (75.8 ± 3.7 pA; *P *< 0.01, one way ANOVA) and a trend toward a lower mean intensity of current injection than IM cells (97.4 ± 7.6 pA; *P = *0.08, one way ANOVA; Fig. 5F). In response to the same intensities of current pulses, RS cells generated the smallest number of spikes with the lowest frequency in both initial and steady state spike firing (Fig. 5A, D) as compared with IM (Fig. 5B, D) and IB (Fig. 5C, D) cells. IB cells exhibit the larger ADP (Fig. 5E; *P *< 0.001, one way ANOVA) as compared with IM cells and RS cells, which facilitated IB cells in generation of an initial doublet of action potentials with high initial frequency (Fig. 5C, D). At the same current injection, IB cells also showed the highest frequency in steady state spike firing and generated highest number of spikes among those three groups of cells (Fig. 5A-D).

For properties of single action potential, the half width of the action potential of RS cells (Fig. 5G, 1.66 ± 0.07 ms) was significantly wider than that in IB neurons (Fig. 5G, 1.43 ± 0.04 ms, P < 0.05, one way ANOVA) and in IM cells (Fig. 5G, 1.46 ± 0.05 ms, P < 0.01, one way ANOVA). IB cells (see Table 1) also showed faster kinetics with decay time (1.53 ± 0.08 ms) shorter than either RS (2.09 ± 1.12 ms, *P *< 0.001, one way ANOVA) cells or IM cells (1.63 ± 0.06 ms, *P *< 0.01, one way ANOVA).

### Intrinsic properties of ACC pyramidal neurons after neuropathic pain

To investigate whether or not the intrinsic properties of ACC pyramidal neurons are altered after nerve injury, we studied the firing patterns and action potentials in mice after undergoing neuropathic pain. Animals with the sham surgery were used as the control. Recordings were performed between 7-14 days after the nerve injury, a time when the maximal behavioral sensitization can be observed [24]. Before electrophysiological experiments, behavioral allodynic responses were always evaluated. First, we found that there was no significant change the in proportion of cell types in the ACC of neuropathic pain mice as compared with control mice. Similar percentages were found among IM cells (neuropathic: 47.5% V.S. control: 44.4%), IB cells (neuropathic: 31.2% V.S. control: 30.9%) and RS cells (neuropathic: 21.3% V.S. control: 24.7%).

**Figure 6:**
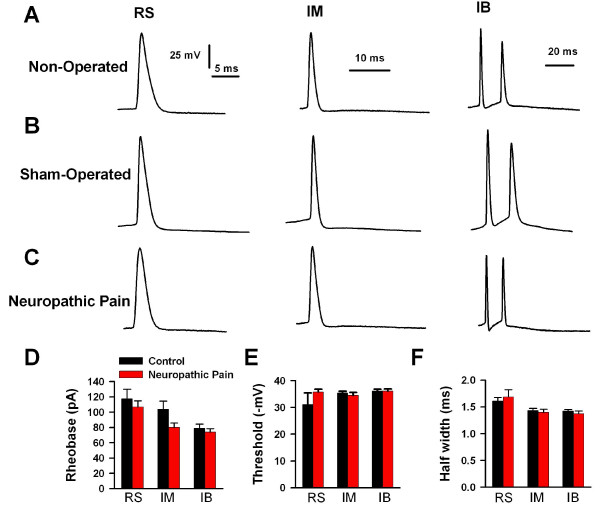
**Changes of action potential properties of IM pyramidal neurons in the ACC after neuropathic pain**. A-C, Examples of APs generated from RS, IM, and IB neurons. All responses elicited in response to a 400 ms depolarizing current pulse of appropriate suprathreshold magnitude. Current records omitted for clarity. A, APs of neurons from non-operated mice; B, APs of neurons from sham-operated mice; C, APs of neurons from neuropathic pain mice; D-F, Histograms for data from RS (control, n = 20; neuropathic, n = 13), IM (control, n = 36; neuropathic, n = 29), and IB (control, n = 25; neuropathic, n = 19) neurons reflect rheobase, AP threshold and half width from control mice (non-operated and sham-operated) and neuropathic pain mice (P > 0.05, Student *t*-test). All values are given as the mean ± SEM.

**Figure 7:**
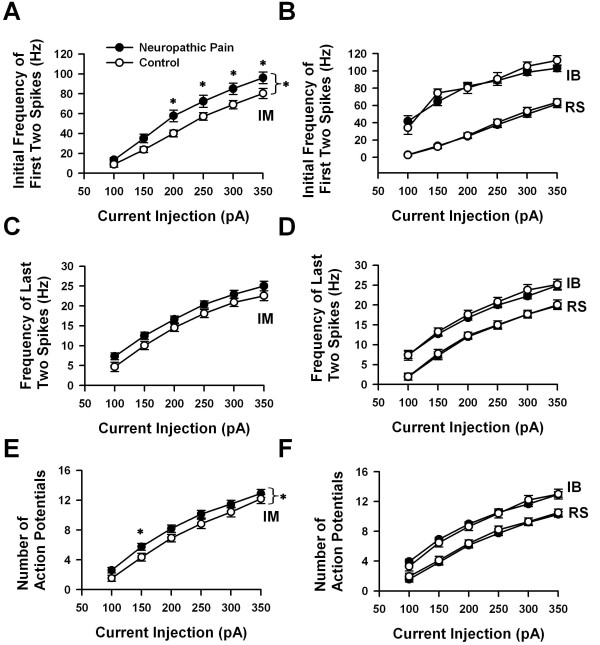
**Summary of alterations seen in firing patterns in ACC pyramidal neurons after neuropathic pain**. A-B, Plot of the initial discharge frequency measured from the 1^st ^interspike interval at different stimulation intensities of depolarizing current (100--350 pA, 400 ms). C-D, Plot of the steady-state discharge frequency measured from the last interspike interval at different stimulation intensities of depolarizing current (100--350 pA, 400 ms). E-F, Plot of the number of APs at different stimulation intensities of depolarizing current (100--350 pA, 400 ms); (A), (C) and (E) show relationships for IM neurons; (B), (D) and (F) for RS and IB neurons. Note that in IM cells, the initial discharge frequencies (*P < 0.05, *F*_1,389 _= 23.34, two-way ANOVA) and the number of APs (*P < 0.05, *F*_1,389 _= 13.47, two-way ANOVA) attained in neuropathic pain cells are greater than those seen in controls. Error bars represent S.E.M. Control data was collected from 20 RS, 36 IM and 25 IB neurons. Neuropathic pain data was collected from 13 RS, 29 IM and19 IB cells, respectively.

Next, we compared the passive membrane and the single action potential properties. Figure 6A-C show representative recordings of APs elicited in RS, IM, and IB neurons by a 400 ms depolarizing current. There were no significant differences in parameters tested, such as RMP, membrane resistance, current threshold, action potential amplitude, half width, rise time and decay time (Fig. 6D-E, see Table 1). Finally, we compared the firing pattern of these three types of pyramidal cells in neuropathic and control animals (Fig. 7). Membrane excitability of RS and IB neurons from neuropathic or control animals was indistinguishable (Fig. 7B, D, F). For IM cells, however, with the same current injection, neuropathic pain cells (n = 29 cells) showed higher initial frequency compared with control cells (n = 36, p < 0.05, two-way ANOVA; Fig. 7A). No difference was found for the steady state frequency of IM cells between neuropathic and control mice (Fig. 7C). Moreover, neuropathic pain cells (n = 29) produced more spikes for the same current injection compared with control cells (n = 36, p < 0.05, two-way ANOVA; Fig. 7E).

## Discussion

The present study is the first to systematically characterize the action potential properties of ACC pyramidal cells and their alterations after nerve injury in adult mice. Considering the cumulative reports of studies using transgenic and gene knockout mice [27,30], the current study provides important information of intrinsic properties for mouse ACC neurons. Three main electrophysiological classes of ACC cells were distinguished according to their firing pattern: (i) RS cells; (ii) IM cells; and (iii) IB cells. From single labeled cell morphological analyses, we found that these cells send its branches or terminals to layer I as well as layer V/VI, suggesting that they form broad neuronal connections with neurons in superficial and deeper layers within the ACC. Finally, after neuropathic pain, we found that the firing rates in IM neurons were increased compared with control IM cells. Our work provides the basic map for future investigations of molecular mechanism for long-term plastic changes in neuronal properties after nerve injury.

### ACC, synaptic transmission, plasticity and spike

Previous spike studies were recorded from PFC neurons in young rats [31,32] and pyramidal neurons located in layer V, major cortical output neurons to other cortical and subcortical areas. For example, Yang et al. [31] reported that there are four major pyramidal cells found in the Layer V of the rat PFC, regular spiking, intrinsic bursting, repetitive oscillatory bursting and intermediate cells. To our knowledge, the present report is the first systemic studies of pyramidal neurons in the layer II/III of ACC areas. Although the PFC in previous reports often contains some of rostral part of ACC neurons, most of current recordings are performed from neurons located caudal ACC to the typical PFC area. Although many anatomic and functions of the PFC-ACC are similar between rats and mice, we feel that it is necessary to study the adult mouse ACC in a systemic manner. Taking advantage of transgenic and gene knockout mice, recent studies reveal novel molecular and synaptic mechanisms for synaptic transmission and plasticity in the ACC [13,15,27,33,34] For example, using genetic deletion of GluR5, Glur6, or GluR5&6, Wu et al (2005a) demonstrated that glutamate kainate receptor GluR5 and 6 contribute to excitatory synaptic transmission in the synapses of layer II/III ACC. By using AC1 and AC8 gene knockout mice, Zhao et al (2006) and Xu et al (2008) showed that calcium-stimulated AC1 contribute to long-lasting synaptic changes within the ACC after peripheral inflammation or nerve injury. These studies would be impossible using traditional pharmacological methods, since there is no selective inhibitor for these target proteins. Unlike synaptic transmission and plasticity in the ACC, little work has been done on spike analyses in the ACC. In the present studies we focused on layer II/III, because (i) our previous work has mostly focused on synaptic transmission and plasticity of ACC layer II and III; (ii) most of cells located in layer II/III are pyramidal cells; (iii) neurons in layer II/III receive sensory inputs [27,34]; and finally, (iv) neurons in the layer II/III are activated by peripheral sensory stimuli and injury [14,35]. Our current spike work is the first study in adult mouse ACC, and provides basic information about the intrinsic properties of cingulate pyramidal cells. We are currently studying inhibitory neurons in the layer II/III as well as neurons located in other layers of the ACC in adult mice.

### Classification of three types of ACC pyramidal cells

This study has identified three main types of pyramidal cells in adult mouse ACC, based on their specific discharge patterns in response to depolarizing current pulses. To our knowledge, this is the first report in the ACC region. The classification of the different electrophysiological types of ACC pyramidal cells proposed in the present study has taken into account the previously reported classifications of cortical pyramidal cells. The three types of cells are mainly classified by whether they can generate burst and whether there is ADP following their first action potential. In young rat PFC, previous studies have revealed four classes of pyramidal neurons, mainly on the basis of their response to application of prolonged intracellular current pulses and the shape of their action potentials [31]. As compared with rat PFC, we have found mostly similar results in adult mouse ACC. There are three major classes of pyramidal cells found in rats and mice, RS, IB and IM. In rat PFC region, Yang et al (1996) identified repetitive oscillatory bursting cells (ROB) at about 13% of the total cells recorded. However, we did not detect any ROB cell in mouse ACC so far. One possible explanation is the region and animal difference, since previous reports were from layer VI of the rat PFC. It will be important to investigate if ROB cell may be found in the layer VI of the mouse ACC in future studies.

### Synaptic and nonsynaptic plasticity

Recent studies have indicated that sensitization at different levels along somatosesnory pathways are likely contributing to chronic pain including neuropathic pain; these include sensitization at peripheral, spinal cord, amygdala and cortex such as ACC [27,34,36,37]. At sensory synapses between afferent fibers and dorsal horn neurons, it has been believed that LTP as well as heterosynaptic facilitation are triggered by peripheral injury [38,39]. Similar LTP were proposed in the amygdala and ACC. In the amygdala, it has been reported that peripheral inflammation or nerve injury triggered long-lasting changes in excitatory synaptic transmission in the central nucleus of the amygdala [37]. In the ACC, Zhao et al (2006) and Xu et al (2008) reported that peripheral nerve injury or inflammation caused long-lasting, LTP-like synaptic changes in the ACC layer II/III synapses [16,17]; and both presynaptic increases of glutamate releases and enhancement of postsynaptic AMPA receptor mediated responses are thought to contribute to the injury-triggered potentiation.

In addition to synaptic plasticity, it has been noted that nonsynaptic plasticity may also play important roles in learning, memory and other brain functions [40-43]. Typically, such nonsynaptic plasticity contains the changes in spike threshold, spike accommodation, amplitude of burst-evoked after hyperpolarization. It has been reported that learning produced long-lasting changes in the intrinsic excitability of central neurons that are known to contribute to the behavioral learning tasks such as cortical, hippocampal and cerebellum neurons [40]. A recent study nicely showed that different neurotransmitter receptors and intracellular signaling pathways are contributing to synaptic potentiation and nonsynaptic plasticity in the subicular pyramidal neurons, respectively [43]. In sensory or nociceptive system, it has been known for many years that peripheral tissue or nerve injury triggered enduring changes in neuronal spike responses to sensory stimulation [44-46]. However, most of previous studied did not distinguish the contribution of synaptic plasticity (e.g., LTP) and/or intrinsic plasticity [40] to such increases, in part, due to the limit of recording technique (i.e., extracellualr recordings in vivo).

However, some important findings have been reported in the DRG cells, first sensory neurons in the CNS. It has been reported that rat nerve injury tended to reduce rheobase and increase the number of APs in response to the same depolarizing currents injection, the AP amplitude and spike width [47-49], many of which mimic those nonsynaptic plasticity found in learning models [40]. In amygdala, the neurons from both visceral pain model and arthritis pain model showed an increased action potential firing rate compared with control neurons [50,51]. In the present study, we have found that nerve injury induced an increased firing rate in IM cells, suggesting that not all neurons are involved in processing the injury information by changes in intrinsic properties. The alterations of the expressions and/or properties of ion channels underlying the action potentials may account for the changes of firing rates. In summary, the results of this study suggest that the plastic change of intrinsic plasticity of ACC neurons is involved in the central processing of neuropathic pain.

## Methods

### Animal preparation

Adult (6-8 weeks old) male C57BL/6 mice were purchased from Charles River. Mice were maintained on a 12 h light/dark cycle. Food and water were provided *ad libitum*. Experiments were performed under protocols approved by the University of Toronto Animal Care Committee. A model of neuropathic pain was induced by the ligation of the common peroneal nerve (CPN) as previously described [24]. Briefly, mice were anaesthetized by intraperitoneal injection of a mixture saline of ketamine (0.16 mg/kg, Bimeda MTC, Cambridge, Ontario) and xylazine (0.01 mg/kg, Bayer, Toronto, Canada). 1 cm skin incision was made in the left hind leg to expose the CPN. The CPN was ligated with chromic gut suture (5-0, Ethicon, Somerville, New Jersey) slowly until contraction of the dorsiflexors of the foot was visible as twitching of the digits. Sham surgery was conducted in the same manner but the nerve was not ligated. The mechanical allodynia was tested on post-surgical day 7 and the mice were used for electrophysiological studies on post-surgical days 7-14.

### Whole-cell patch-clamp recording

The animals were decapitated and the brain was quickly removed and immersed in oxygenated (95% O_2_-5% CO_2_) cooled (4~6°C) artificial cerebrospinal fluid (ACSF) for 2-3 minutes. ACSF contained (in mM): 124 NaCl, 2.5 KCl, 2 CaCl_2_, 2 MgSO_4_, 25 NaHCO_3_, 1 NaH_2_PO_4_, 10 glucose, pH 7.4, 300-310 mOsm. A block of brain tissue containing the ACC was dissected, glued to a small stage (LOCTITE 404 cyanoacrylate glue) and covered with ACSF. Coronal slices 300 microns thick containing ACC were made with a vibratome (Series 1000) and preincubated in oxygenated ACSF at room temperature (22~26°C) for at least 1 hour, then transferred to a submerged chamber and superfused (2~3 ml/min) with oxygenated ACSF at room temperature. Experiments were performed in a recording chamber on the stage of a BX51W1 microscope equipped with infrared DIC optics for visualization. Using the whole-cell patch-clamp technique, recordings were obtained from layer II/III neurons with an Axon 200B amplifier (Axon Instruments, CA). Patch pipettes with resistances of 3~5 MΩ. were filled with the following solution (in mM): 120 K-gluconate, 5 NaCl, 1 MgCl_2_, 0.2 EGTA, 10 HEPES, 2 Mg-ATP, 0.1 Na_3_-GTP and 10 phosphocreatine disodium (adjusted to pH 7.2 with KOH). Biocytin (0.2%) was included in the pipette solution to label recorded neurons. Access resistance <30 MΩ was considered acceptable. Usually, only one neuron per slice was recorded. Data were discarded if access resistance changed more than 15% during an experiment. Data were filtered at 1 kHz, and digitized at 10 kHz.

### Behavioral allodynic responses

Mice were placed in a plexi-glass restrainer and allowed to acclimate for 30 minutes prior to behavioral testing. Mechanical allodynia was assessed based on the responsiveness of the hind paw to the application of von Frey filaments (Stoelting, Wood Dale, Illinois) to the point of bending. Positive responses include licking, biting and sudden withdrawal of the hind paw. Experiments were carried out to characterize the threshold stimulus. Mechanical pressure from a 1.65 filament (force 0.008 g) was found to be innocuous in control mice. This filament was then used to test the mechanical allodynia after neuropathic pain. Ten trials were carried out each time at an interval of 5 minutes and the results are expressed as percentage of positive responses.

### Passive membrane properties

Off-line analysis was performed using Clampfit version 9 (Axon Instruments). Resting membrane potential (RMP) was the low-pass readout of the electrode amplifier and was not corrected for liquid junction potential (~12 mV) after terminating the recording. The membrane potential was measured immediately after establishing the whole-cell configuration. Only neurons that had a resting membrane potential more negative than -60 mV were further investigated. Conductance was determined from the linear slope (between -60 mV to -80 mV) of the current-voltage (I-V; V_hold _= -70 mV) relationships.

### Active membrane properties and firing patterns

Action potentials (APs) were detected in response to suprathreshold current injections from a holding potential around -70 mV. Depolarizing currents of 5~200 pA (400-ms duration) were delivered in increments of 5 pA until an AP was evoked. The rheobase was defined as the minimum current required to evoke an action potential. The AP voltage threshold (V_threshold_) was defined as the first point on the rising phase of the spike at which the change in voltage exceeded 50 mV/ms. The spike amplitude was quantified as the difference between the V_threshold _and the peak voltage. The duration of the AP was measured at the threshold voltage. The spike width was measured at 1/2 of the total spike amplitude (measured from the V_threshold _level). The time to the peak of fast component of the afterhyperpolarization (fAHP) was estimated as the time from the peak of the action potential to the most negative voltage reached during the fAHP (defined as the peak of fAHP). The amplitude of fAHP was estimated as the difference between the V_threshold _and the peak of fAHP. If an afterdepolarization (ADP) was present, its amplitude was determined as a distance between the peak of fAHP to the peak of ADP. Amplitude of slow component of the afterhyperpolarization (sAHP) was measured from the V_threshold _to the peak of sAHP. The time to the peak of sAHP was estimated as the time from the peak of the action potential to the peak of sAHP. The waveform characteristics of the action potentials recorded from neurons of control and neuropathic pain mice, i.e., maximum rise slope, maximum decay slope, rise time, rise slope, decay time and decay slope, were determined using Clampfit 9.2 software (Axon Instruments). The properties of firing patterns and hyperpolarizing responses were analyzed from voltage responses to injected current pulses. Instantaneous firing frequency was calculated as the reciprocal of the interspike interval (ISI). The spike firing frequencies were plotted against the interval number since train onset.

### Histology and immunohistochemistry

After electrophysiological recordings, slices containing biocytin-filled ACC neurons were fixed overnight in a cold solution containing 4% paraformaldehyde. The slices were then collected in 1% stock Tris buffered saline for 1 hour and washed twice (10 minutes each time) in Tris buffered saline. Slices were then incubated in PBS with 0.1% Triton X-100 (JT Baker; Phillipsburg, NJ) for a period of 2 hours to enhance the penetration of the subsequent streptavidin. The biocytin filled cells were rendered fluorescent by incubating overnight in a Cy3-conjugated streptavidin (Jackson ImmunoResearch Labs; West Grove, PA) solution (1 *m*g/ml of PBS) at 4°C. The following day, slices were equilibrated in 1% Tris buffered saline and mounted on glass slides.

### Confocal microscopy

Labeled neurons were imaged by a confocal microscope (Fluoview FV 1000, Olumpus, Tokyo, Japan). Optical sections, usually at consecutive intervals of 1-2 μm, were imaged through the depth of the labeled neurons and saved as image stacks. Collapsing this stack using z projection on the confocal software onto a single plane generated a two-dimensional reconstruction of the labeled neuron. The image stack was also reconstructed in 3-D with appropriate software, to define areas of interest in the neuron. Although the effects of laser illumination on fixed tissue are not known, to prevent possible ultrastructural damage we tried to minimize both the scanning time and the laser intensity. The horizontal extent of axons was measured as the average distance between the three most distal axonal endings on each side from the soma of individual pyramidal neurons. The photomicrograph (Fig. 1C) was assembled by using Adobe Photoshope. Only brightness and contrast were adjusted.

### Data analysis

Results are expressed as means ± SEM. Statistical comparisons were performed using ANOVA, the Student *t*-test and χ^2 ^test. The level of significance was set at *P *< 0.05.

## Abbreviations

ACC: anterior cingulate cortex; RS: regular spiking cells; IB: intrinsic bursting cells; IM: intermediate cells; RMP: resting membrane potential; fAHP: fast afterhyperpolarization; sAHP: slow afterhyperpolarization; ADP: afterdepolarization; APs: action potentials; V_hold_: holding potential; V_threshold_: action potential voltage threshold; ISI: the interspike interval.

## Competing interests

The authors declare that they have no competing interests.

## Authors' contributions

XYC carried out electrophysiological and behavioral experiments and drafted the manuscript. HX, LJW, XL, TC participated in electrophysiological experiments. TC helped with confocal experiments. MZ designed and finished the final draft of the manuscript. All authors read and approved the final manuscript.
